# Biological Sex and Outcomes in Patients with Extracranial Cervical Arterial Dissections

**DOI:** 10.3390/jcm14113816

**Published:** 2025-05-29

**Authors:** Issa Metanis, Naaem Simaan, Yoel Schwartzmann, Tamer Jubeh, Asaf Honig, Hamza Jubran, Jad Magadle, John M. Gomori, Jose E. Cohen, Ronen R. Leker

**Affiliations:** 1 Department of Neurology, Hadassah-Hebrew University Medical Center, P.O. Box 12000, Jerusalem 91120, Israel; issa.meta@yahoo.com (I.M.); naaem.simaan@gmail.com (N.S.); yoelschwartzmann@gmail.com (Y.S.); tamer0001@hotmail.com (T.J.); asaf.honig2@gmail.com (A.H.); hamzeh-89@hotmail.com (H.J.); j.magadle@gmail.com (J.M.); 2 The Azrieli Faculty of Medicine, Bar Ilan University, Ramat Gan 5290002, Israel; 3 Department of Neurology, Ziv Medical Center, Safed 13100, Israel; 4 Department of Neurology & Stroke, Soroka Medical Center, Ber Sheva 84101, Israel; 5 Department of Radiology, Hadassah-Hebrew University Medical Center, P.O. Box 12000, Jerusalem 91120, Israel; gomori@hadassah.org.il; 6 Department of Neurosurgery, Hadassah-Hebrew University Medical Center, P.O. Box 12000, Jerusalem 91120, Israel; jcohenns@yahoo.com

**Keywords:** dissection, arterial, extracranial, biological sex, stroke

## Abstract

**Background and Aims:** Cervical arterial dissections (CeAD) are a common cause of stroke in young adults across both sexes. Whether biological sex plays a role in the pathogenesis and outcome of CeAD remains unclear. **Methods:** In this retrospective analysis of a cohort of patients with CeAD, clinical, imaging, treatment, and outcome data were compared between females and males using multivariate logistic regressions to identify outcome predictors. Propensity score matching (PSM) was used to adjust for imbalances between the groups. **Results:** Overall, 135 participants were included (79 males and 56 females, median age 44, interquartile range [IQR] 36, 50.5). Of those, 71 patients (53%) were diagnosed with stroke (median age 46, IQR 39.5, 52, median admission NIHSS 3, IQR 1, 7.5). Males had significantly higher rates of smoking (38% vs. 11%, *p* = 0.0004) but other baseline characteristics did not differ between the groups. Traumatic dissections were numerically more common in men but the difference between the groups did not reach significance. The presence of flame shaped lesion in the extra cranial vessel was more common among men in the initial analysis of the whole group but did not remain significant after PSM. No differences were observed between the groups regarding treatment strategies including administration of systemic thrombolysis and stent placements. The rates of recurrent stroke and recurrent dissections were similar. Favorable outcomes defined as modified Rankin Score (mRS) ≤ 2 and symptomatic intracranial hemorrhage rates were also similar on the univariate analyses and did not change after PSM. Age (odds ratio [OR] 1.12, 95% confidence intervals [CI] 1.04–1.23) and admission NIHSS (OR 0.74, 95%CI 0.60–0.84) were associated with outcomes on regression analysis whereas female sex was not (OR 0.54, 95% CI 0.03–5.87). **Conclusions:** CeAD occurs more frequently in males, who are more likely to have associated risk factors and traumatic neck injuries. However, sex does not appear to impact outcome in CeAD patients.

## 1. Introduction

Cervical arterial dissections (CeAD) are common in young adults and can lead to significant morbidity including stroke, isolated cranial nerve injuries, and severe headaches [1,2,3,4,5]. CeAD accounts for up to 25% of all strokes in young adults and is not infrequently misdiagnosed especially if the initial symptoms are less specific and the patients are present with headache, isolated cranial nerve injuries, or isolated Horner’s syndrome. CeAD occurs in both men and women and can be classified as traumatic (tCeAD) or spontaneous (sCeAD) dissections [2]. There are several potential differences in the pathogenesis of dissection between men and women, including differences in the prevalence of collagen disorders, genetic risk factors for dissections, and hormonal factors. Previous studies suggested that CeAD may have different etiologies in men and women which may lead to different outcomes [6,7,8,9,10] and dictate different therapeutic interventions. For example, the risk of stroke complicating CeAD was reported to be higher in men [9,10,11], whereas the risk of recurrent dissections was reportedly higher in women [9], leaving significant knowledge gaps that need to be explored. Two previous studies found a higher incidence of CeAD in men with similar clinical manifestations and outcomes between men and women [12,13]. However, these studies included both extracranial and intracranial dissections and only one of those previous studies also included patients with traumatic dissections [13], which are not uncommon in patients with CeAD. Whether dissections differ between men and women in clinical, radiological and treatment or outcome characteristics remains largely unexplored. Therefore, we aimed to compare data from patients from both biological sex who suffered from CeAD in a more inclusive dataset that focused on extracranial dissections and included all patients with sCeAD and tCeAD.

## 2. Materials and Methods

This was a retrospective case series study of patients with CeAD identified from a large cohort of stroke patients admitted to a tertiary teaching hospital over the span of six years (2016–2022). The study was approved by the institutional ethics committee with an exemption from obtaining informed consent due to the retrospective use of anonymized data. This report follows STROBE guidelines. The diagnosis of CeAD was confirmed by a senior neuro-radiologist on imaging studies including CT angiography, MR angiography, or digital subtraction angiography showing evidence of extracranial intimal flap, double lumen, flame shaped occlusions, progressive narrowing of the vessel, or tapering of the vessel lumen leading to more distal occlusion. Patients with isolated intracranial dissections were excluded but extension of the dissecting flap from the extracranial vessels into the intracranial vasculature was allowed. Patients with or without stroke were included and the only inclusion criteria was the presence of CeAD on imaging. Patients with acute focal neurological symptoms and signs lasting more than 24 h or patience present with acute focal brain lesions compatible with stroke on imaging were diagnosed with stroke. In contrast, those without acute or late (within a few days) imaging confirmation of stroke (e.g., patients presenting with isolated cranial nerve injury, isolated tinnitus or Horner sign) were categorized as non-stroke patients. Data were initially analyzed for the entire cohort regardless of the diagnosis of stroke complicating CeAD. On a secondary analysis, we separately analyzed data of patients that were discharged with a diagnosis of stroke during the same admission as that for CeAD. Included patients were divided according to biological sex. Sex was defined according to reported sex in the medical records. CeAD were further divided into spontaneous (sCeAD) and traumatic (tCeAD) in accordance with previous definitions [2]. Briefly, traumatic dissections included any direct mechanical impact or injury to the head and/or neck and any activity involving prolonged increased thoracic pressure. These events had to occur less than 30 days from diagnosis of dissection to qualify as tCeAD.

Data were collected from electronic medical files including outpatient visits to the neurology and neurosurgery clinics. We collected demographics, stroke-risk factor profile, clinical stroke characteristics, severity, and imaging findings. All patients underwent non-contrast CT scans and CT angiography, and some also had a CT perfusion study. Degree of stenosis of the dissected artery was categorized as none (0% narrowing), mild (<50% narrowing), moderate (50–70% narrowing), severe (70–99% narrowing), and vessel occlusion (100% narrowing). Evaluations of vessel recanalization were performed on follow up vascular imaging studies, obtained either during admission or within the following 30 days.

We also collected information on the treatments given and outcome parameters. The primary outcome for this study was favorable functional outcome defined as modified Rankin scale (mRS) of ≤2 at 90 days post CeAD and stroke. Other outcome parameters included excellent outcome at day 90 post CeAD defined as mRS ≤ 1, vessel recanalization rates on follow-up imaging studies and the presence of any intracranial hemorrhage (ICH) or symptomatic ICH (sICH) defined as an ICH leading to new neurological symptoms with NIHSS worsening by 4 points or more. Survival and rates of recurrent stroke and recurrent dissection at the time of the last follow-up meeting were documented.

### Statistical Analysis

Statistical analysis was conducted using R version 4.4.1 (R Foundation for Statistical Computing, Vienna, Austria). All analyses were performed by a professional biostatistician (see acknowledgments). We analyzed patient characteristics with categorical variables summarized as counts and percentages and compared using the χ^2^ test or Fisher exact test. Analysis of continuous data are presented as mean ± standard deviation (SD) or as median with interquartile range (IQR). A *p* < 0.05 was considered significant. Due to the non-normal distribution of these variables, differences between genders were assessed using the Mann–Whitney U test, which highlighted notable disparities, particularly higher morbidity among males.

To mitigate these imbalances, propensity score matching (PSM) was implemented using the optimal method with a caliper setting of 0.05. Adjustments were made for variables including age at diagnosis, lipid levels, smoking status, isolated neck injury, initial NIHSS score, pre-stroke mRS, vessel stenosis, presence of a double lumen, occlusion above the bifurcation, and flame-shaped occlusions.

Logistic regression was applied to examine whether gender significantly affects the likelihood of a favorable outcome at 90 days. Due to the low number of unfavorable outcomes, we could only include a limited number of variables in this analysis in addition to the mandatory inclusion of sex. Therefore, we decided to include variables that are well recognized as important predictors of outcome including age and stroke severity with mandatory inclusion of sex. In a secondary exploratory analysis, we also included other variables that yielded a *p* < 0.05 in the univariate analysis.

## 3. Results

Among the 154 patients with CeAD that were screened for the study, 93 (60%) were male and 61 (40%) were female. We excluded three patients due to an admission NIHSS score of 42 due to deep coma, eight due to missing mRS data at 90 days, and another eight due to a pre-stroke mRS score greater than 2, resulting in a total of 135 participants—79 males and 56 females that were included (Supplementary Figure S1). The median age of the included patients was 44 (IQR 36, 56) and the median admission NIHSS for the entire group was 0 (IQR 0, 4). Overall, 71 patients (53%) were diagnosed with stroke (median age 46, IQR (39.5, 52)) with a median (IQR) admission NIHSS scale of 3 (1, 8).

In the primary analysis that included all patients with CeAD (Table 1), males were more often smokers (38% vs. 11%, *p* = 0.0004) but other baseline characteristics did not differ between the groups. The carotid artery was the most commonly involved artery in both men and women and in most cases, the dissection started at the extracranial carotid bifurcation. To control for potential imbalances between the groups we performed a propensity score analysis of the data that showed no differences between men and women in any of the baseline characteristics. In the secondary analysis that was limited to patients that suffered a stroke, hyperlipidemia was the only parameter that differed between the groups (26% vs. 4%, in men vs. women, respectively; *p* = 0.007). Connective tissue disorders were recorded in 13 (8%) patients overall (9 with fibromuscular dysplasia, 2 with Ehlers Danlos syndrome and 1 each with Marfan and Loeys-Dietz syndromes). Overall, these connective tissue disorders were more common in women (10/56 [16%] vs. 3/79 [4%], *p* < 0.001). Analysis of the whole group showed that the initial neurological disability did not significantly differ between men and women (Table 1). However, on the secondary analysis that only included patients with stroke, the initial stroke severity was significantly lower in females (admission NIHSS median, (IQR); 2 (0, 4) vs. 4 (2, 14), *p* = 0.015; Supplementary Table S1). This difference remained significant after PSM (Supplementary Table S1). The presence of flame shaped lesion in the extra cranial vessel was more common among men in the initial analysis of the whole group but did not remain significant after PSM. Other imaging parameters used for diagnosing CeAD dissections did not differ between the groups (Table 1). These findings were similar for the secondary analysis of patients with stroke (Supplementary Table S1). The severity of stenosis was higher in men with significantly higher rates of vessel occlusions observed among men (Table 1). These findings showed comparable trends in patients that had a stroke but missed statistical significance (Supplementary Table S1). Post-matching analysis revealed no significant differences in baseline characteristics between males and females (Table 1).

Treatment strategies including the use of systemic thrombolysis and the use of stenting were similar between the groups (Table 1). Notably, favorable target vessel recanalization to thrombolysis in cerebral infarction 2b-3 rates were also similar. Corresponding results were observed in the secondary analysis of patients with stroke (Supplementary Table S1).

Intracerebral hemorrhages were seen in 10 patients overall with no significant differences between men and women (Table 2). Notably, all tCeAD patients with ICH (n = 7) had mixed types of hemorrhages including intracerebral, subarachnoid, and subdural hemorrhages secondary to the traumatic brain injury. Most patients with sCeAD and ICH (n = 3) had coexisting subarachnoid hemorrhages seen as complications of stenting with intracranial extension of the dissection (2/3 [67%]).

**Figure 1:**
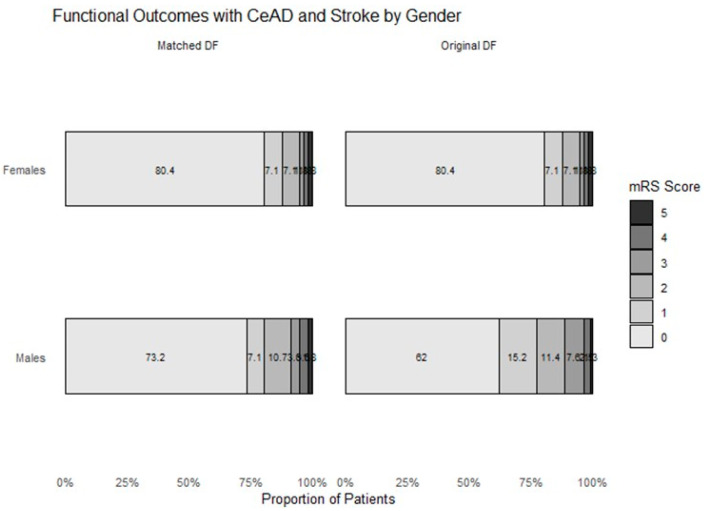
Bar graph plots depicting functional outcomes measured with the modified Rankin Scale in patients with CeAD and stroke by sex 90 days post admission before (**right panels**) and after (**left panels**) propensity score matching. Numbers represent percentages of patients in each category.

Data on outcome obtained 90 days post-presentation was available for all 135 of the included patients. Similarly, in the secondary analysis that only included patients with stroke, follow up data were available for all patients. Vascular imaging during follow up was obtained for most patients and was obtained at a median of 1 month (IQR 0, 3) from initial admission. The rates of recurrent dissections and recurrent stroke were very low and did not differ between the groups. Many patients were initially treated with therapeutic doses of low molecular weight heparin in both groups, but the treatment was usually given only in the first week or two and then the patients were switched to antiplatelet agents without differences between the groups. Long-term preventive treatment after discharge mostly consisted of antiplatelet agents in both men and women with a median length of treatment of 13 months (IQR 10, 20) and did not differ between the groups. On day 90, the rates of functional independence were similar (95% vs. 88%, in female vs. male patients, respectively, *p* = 0.358) with similar rates of recurrent stroke and recurrent dissections (Table 2). Excellent outcome rates (mRS 0–1) did not significantly differ between the groups (89% vs. 80%, *p* = 0.161). These results did not change after PSM (Table 2). Likewise, in the analysis that included only patients with stroke, there were no statistically significant differences in functional independence rates on day 90 with numerically higher rates of independence observed in females (93% vs. 88% *p* = 0.453; Figure 1 and Supplementary Table S2). Analysis of the data for excellent outcomes among patients with stroke also failed to show statistically significant differences between men and women despite numerically higher chances for excellent outcomes in women (83% vs. 66%, *p* = 0.114). Post-matching analysis revealed no significant differences in 90-day mRS outcomes between males and females (Supplementary Table S2).

Variables associated with favorable outcomes were then investigated (Table 3). This analysis showed that patients with favorable outcomes were significantly older and had lower initial stroke severity (Table 3). Patients with favorable outcomes also less often had multi-vessel involvement or symptomatic intracerebral hemorrhage and more often had elongated stenosis (Table 3). Stents were more often placed in patients with unfavorable outcomes (Table 3).

We next performed a regression analysis controlling for biological sex, age, and admission NIHSS (Table 4). Due to the low number of patients with unfavorable outcomes, we could include a limited number of variables in the regression. In this analysis, age (odds ratio [OR] 1.12 95% confidence intervals [CI] 1.04, 1.24) and admission NIHSS (OR 0.73, 95%CI 0.60, 0.84) were associated with outcomes, whereas sex was not (OR 0.53, 95% CI 0.03, 5.80). In a secondary exploratory analysis that also included the presence of multi-vessel dissections, vessel occlusions, and sICH in addition to age, NIHSS and gender (Supplementary Table S3) sex also did not affect outcomes.

## 4. Discussion

The main findings of the current analysis are that CeAD are more common in males and that despite higher rates of smoking and isolated neck injury in males, biological sex does not correlate with the chances of achieving favorable outcomes.

Several potential mechanisms could contribute to differences in pathophysiology and outcomes between males and females with CeAD. For example, connective tissue disorders could be over-represented in females and could theoretically have an impact on outcomes and treatment [14,15,16,17]. In contrast, fibromuscular dysplasia was found to be associated with dissection and stroke more often in males [7]. Additionally, hormonal changes may affect arterial stiffness which is known to affect the occurrence and recovery of CeAD [18]. Arterial stiffness is often more severe in females [19] and may thus lead to less favorable outcomes in females. Furthermore, genetic factors also play a role in CeAD in both men and women and specific genetic variations may influence the likelihood of CeAD occurrence and recurrence and affect recovery [17,20,21]. These factors could potentially explain the observation of higher stroke rates documented in men with CeAD [9,10,11]. This could potentially lead to poor outcomes predominantly in men. Moreover, inflammation and autoimmunity, which are more common in women, were identified as contributing factors in the development of CeAD [22].

In the current study, we were not able to document differences in outcomes between men and women with CeAD. This may be explained by a similar prevalence of stroke in males and females in the current study. The discrepancy between the current findings and those observed in the previous studies could be explained by the different inclusion criteria used in the studies. Thus, the current study included patients with both tCeAD and sCeAD including patients with severe tCeAD, whereas two of the previous studies only included patients with sCeAD [10,11] and the one study that included patients with tCeAD did not report on the severity or cause of tCeAD [9]. Similar to our findings the long-term functional outcome did not differ between men and women in the latter study [9].

On regression analysis, age, and the degree of neurological impairment upon presentation were found to impact the likelihood of favorable outcomes whereas sex did not. In an expanded regression analysis model age, severity of the initial neurological deficit, presence of vessel occlusion and presence of multiple dissections were identified as modifiers of outcome whereas sex was not. However, given the low number of poor outcomes observed, the results of the latter analysis should be interpreted with caution and need reaffirmation in a larger sample size.

In the current dataset, males more often had tCeAD and more often had complete vessel occlusions, which are correlated with poor outcomes in patients with CeAD [4,23]. However, the rates of severe poly-trauma, which may have negative effects on functional outcomes, did not significantly differ between men and women, which may explain the similar outcomes observed overall. Treatment choices including systemic thrombolysis and stent placement did not differ between males and female patients and did not have an impact on outcome. Other known modifiers of outcome measures such as sICH and recanalization rates also did not differ between men and women. In addition, the rates of recurrent stroke and recurrent dissections were not significantly different between men and women, negating the possibility that the lack of influence of sex on outcome stems from unexpectedly high rates of dissection recurrence or stroke in one sex. Taken together, these findings suggest that biological sex does not have a substantial influence on the outcomes among patients with CeAD.

The current observations are in agreement with two other larger studies that reported higher incidence of CeAD in males and similar clinical manifestations and outcomes between the biological sex [12,13]. However, it is important to note that our study specifically focused on extra-cranial dissections, distinguishing it from previous research which included both extracranial and intracranial dissections. Furthermore, in the current study, we included all patients with CeAD, including those with more severe trauma that were not included in prior studies. Therefore, our results could be viewed as more generalizable to everyday practice and complimentary to the previous set of results.

Our study had several limitations. First, the retrospective design and the fact that this was a single center study might be sources of potential bias including selection bias and bias by indication. However, we used PSM to control for imbalances between the groups which likely mitigates some of these weaknesses. Second, we excluded all patients with isolated intracranial dissections, which limits the generalizability of our results to only patients with extracranial dissections. Third, the study only included a limited number of patients, limiting us from drawing conclusive results. This is especially true when considering the small amounts of patients with specific predisposing conditions such as Ehler–Danlos or Marfans’ disease. However, CeAD is not very common, and we did use an all-inclusive design of all patients with spontaneous and traumatic extracranial dissections, including patients with severe forms of trauma that were mostly excluded from prior studies. Moreover, we had a high follow-up rate of 95% at 90 days post-presentation. Fourth, to the best of our knowledge, no transgender or intersex people were included in the current study. Therefore, our results cannot be generalized to include these populations. Given the large confidence interval observed for the effects of sex on the outcome, the current results should be interpreted with caution and the subject should be studied further in larger registry studies.

In conclusion, the current findings suggest that outcomes in patients with CeAD appear to be modified by age and the severity of the neurological deficits but not by biological sex.

## Figures and Tables

**Table 1:** Baseline characteristics of patients with Cervical arterial dissections.

Variable/Group	Unmatched Patients, No, (%)		*p*	After Propensity Score Matching, No. (%)		*p*
	Males	Females		Males	Females	
	(n = 79)	(n = 56)		(n = 56)	(n = 56)	
Age (Median; Q1, Q3)	45 (36, 51.5)	41 (36.5, 48.2)	0.439	44 (35, 52.2)	41 (36.5, 48.2)	0.852
Hypertension (%)	18 (22.8)	10 (17.9)	0.525	8 (14.3)	10 (17.9)	0.797
Diabetes (%)	5 (6.3)	2 (3.6)	0.699	3 (5.4)	2 (3.6)	1.000
Atrial fibrillation (%)	3 (3.8)	0 (0)	0.266			
Hyperlipidemia (%)	15 (19)	2 (3.6)	0.266	6 (10.7)	2 (3.6)	0.270
Smoking (%)	30 (38)	6 (10.7)	0.0004	14 (25)	6 (10.7)	0.082
Old stroke (%)	3 (3.8)	1 (1.8)	0.641	0 (0)	1 (1.8)	1.000
Migraine (%)	1 (1.3)	4 (7.1)	0.159	1 (1.8)	4 (7.1)	0.363
Violent cough (%)	7 (8.9)	6 (10.7)	0.772	7 (12.5)	6 (10.7)	1.000
Spontaneous dissection (%)	50 (63.3)	43 (76.8)	0.130	36 (64.3)	43 (76.8)	0.213
Neck injury (%)	15 (19)	4 (7.1)	0.077	10 (17.9)	4 (7.1)	0.151
Poly-trauma (%)	14 (17.7)	6 (10.7)	0.328	10 (17.9)	6 (10.7)	0.418
Trauma severity (%)			0.118			0.154
Non Trauma	50 (63.3)	46 (82.1)		36 (64.3)	46 (82.1)	
Mild	12 (15.2)	3 (5.4)		9 (16.1)	3 (5.4)	
Moderate	7 (8.9)	3 (5.4)		4 (7.1)	3 (5.4)	
severe	10 (12.7)	4 (7.1)		7 (12.5)	4 (7.1)	
Premorbid mRS ≤ 2 (%)	78 (98.7)	56 (100)	1.000	56 (100)	56 (100)	1.000
Admission NIHSS (Median; Q1,Q3)	0.0 (0.0, 5.0)	0.0 (0.0, 2.0)	0.070	0 (0.0, 3.0)	0 (0.0, 3.0)	0.326
**Radiological findings (%)**						
Early infarct signs on initial CT/MR	30 (38)	20 (35.7)	0.857	19 (33.9)	20 (35.7)	1.000
Carotid only	48 (60.8)	28 (50)	0.223	34 (60.7)	28 (50)	0.342
Vertebral only	19 (24.1)	18 (32.1)	0.331	14 (25)	18 (32.1)	0.530
More than one vessel	16 (20.3)	13 (23.2)	0.677	11 (19.6)	13 (23.2)	0.818
Intimal flap	17 (21.8)	11 (19.6)	0.831	13 (23.6)	11 (19.6)	0.650
Pseudo-aneurysm	18 (23.7)	20 (35.7)	0.173	10 (18.9)	20 (35.7)	0.056
Mural hematoma	12 (15.2)	14 (25)	0.186	7 (12.5)	14 (25)	0.145
Long stenosis	33 (41.8)	29 (51.8)	0.294	26 (46.4)	29 (51.8)	0.705
Double Lumen	3 (3.8)	6 (10.7)	0.162	3 (5.4)	6 (10.7)	0.489
Vessel occlusion	26 (32.9)	9 (16.1)	0.030	13 (23.2)	9 (16.1)	0.476
Elongated tapering	1 (1.3)	1 (1.8)	1.000	1 (1.8)	1 (1.8)	1.000
Flame shaped	30 (38)	5 (8.9)	0.0001	13 (23.2)	5 (8.9)	0.069
**Treatments (%)**						
tPA	6 (7.6)	2 (3.6)	0.468	1 (1.8)	2 (3.6)	1.000
Stent placement	32 (40.5)	17 (30.4	0.276	18 (32.1)	17 (30.4)	1.000

mRS—modified Rankin scale, NIHSS—National Institutes of Health Stroke Scale, IQR—interquartile range, tPA—tissue Plasminogen Activator.

**Table 2:** Primary and secondary outcomes among patients with dissections.

	Unmatched Patients, No, (%)		*p*	After Propensity Score Matching, No. (%)		*p*
	Males	Females		Males	Females	
	(n = 79)	(n = 56)		(n = 56)	(n = 56)	
ICH (%)	8 (10.1)	2 (3.6)	0.194	7 (12.5)	2 (3.6)	0.161
s-ICH (%)	4 (5.1)	1 (1.8)	0.402	4 (7.1)	1 (1.8)	0.363
Recurrent stroke (%)	6 (7.6)	5 (8.9)	0.762	3 (5.4)	5 (8.9)	0.716
Recurrent dissection (%)	3 (3.9)	0 (0)	0.273	2 (3.7)	0 (0)	0.495
Favorable recanalization (%)	36 (59)	22 (51.2)	0.547	26 (61.9)	22 (51.2)	0.383
mRS on day 90 (%)			0.251			0.926
0	49 (62)	45 (80.4)		41 (73.2)	45 (80.4)	
1	12 (15.2)	4 (7.1)		4 (7.1)	4 (7.1)	
2	9 (11.4)	4 (7.1)		6 (10.7)	4 (7.1)	
3	6 (7.6)	1 (1.8)		2 (3.6)	1 (1.8)	
4	2 (2.5)	1 (1.8)		2 (3.6)	1 (1.8)	
5	1 (1.3)	1 (1.8)		1 (1.8)	1 (1.8)	
mRS day 90 ≤ 2 (%)	70 (88.6)	53 (94.6)	0.358	51 (91.1)	53 (94.6)	0.716
mRS day 90 ≤ 1 (%)	63 (79.7)	50 (89.3)	0.161	46 (82.1)	50 (89.3)	0.418

ICH—Intracerebral hemorrhage, sICH—symptomatic ICH, mRS—modified Rankin scale.

**Table 3:** Comparison of patients with favorable (mRS ≤ 2) and unfavorable (mRS ≥ 3) outcomes.

Variable/Group	Favorable (n = 123)	Unfavorable (n = 12)	*p* Value
Age (median; IQR)	44 (37.5, 52.5)	36 (19.8, 41.3)	0.008
Biological sex (male, %)	71 (57.7)	9 (75)	0.182
Hypertension (%)	27 (22)	1 (8.3)	0.459
Diabetes (%)	7 (7.5)	0 (0)	1.000
Atrial fibrillation (%)	3 (2.4)	0 (0)	1.000
Ischemic heart disease (%)	3 (2.4)	0 (0)	1.000
Hyperlipidemia (%)	16 (13)	1 (8.3)	1.000
Current smoker (%)	34 (27.6)	2 (16.7)	0.514
Prior stroke (%)	3 (2.4)	1 (8.3)	0.314
Early signs of infarct on imaging (%)	45 (36.6)	5 (41.7)	0.760
Involved vessels:			
Carotid only (%)	72 (58.5)	4 (33.3)	0.128
Vertebral only (%)	34 (27.6)	3 (25)	1.000
More than one vessel (%)	23 (18.7)	6 (50)	0.021
Radiological findings:			
Intimal flap (%)	28 (21)	3 (25)	1.000
Pseudo aneurysm (%)	36 (29.8)	2 (18.2)	0.510
Mural hematoma (%)	23 (18.7)	3 (25)	0.700
Elongated stenosis (%)	60 (48.8)	2 (16.7)	0.037
Double lumen (%)	9 (7.3)	0 (0)	1.000
Occlusion (%)	29 (23.6)	6 (50)	0.078
Admission NIHSS (median, IQR)	0 (0–3)	14 (7, 19)	<0.001
tPA (%)	8 (6.5)	0 (0)	1.000
Stent placement (%)	41 (33.3)	8 (66.7)	0.029
sICH (%)	3 (2.4)	3 (25)	<0.001
Recurrent dissection (%)	2 (1.6)	1 (8.3)	0.130
Favorable recanalization (%)	54 (43.9)	6 (50)	0.721

NIHSS—National Institutes of Health Stroke Scale, IQR—interquartile range, mRS = modified Rankin Scale, tPA—tissue Plasminogen Activator, sICH—symptomatic intracranial hemorrhage.

**Table 4:** Factors associated with favorable (mRS ≤ 2) outcome at 90 days post stroke on logistic regression.

Variable/Group	OR	95% CI	*p*
Age (per year)	1.12	1.04–1.24	0.008
Biological sex (male)	0.53	0.03–5.80	0.614
Admission NIHSS	0.73	0.60–0.84	<0.001

NIHSS—National Institutes of Health Stroke Scale. mRS = modified Rankin Scale.

## Data Availability

Data pertinent to this study will be made available upon request and in line with regulations imposed by governmental and intuitional policies.

## Supplementary Materials

The following supporting information can be downloaded at: https://www.mdpi.com/article/10.3390/jcm14113816/s1. Supplementary Figure S1: Patient flow chart; Table S1: Baseline characteristics in patients with CeAD and stroke based on Gender; Table S2: Treatments and Outcomes in patients with CeAD and stroke based on Gender; Table S3: Factors associated with favorable (mRS ≤ 2) outcome at 90 days post stroke on logistic regression.

## Abbreviations

CeAD	cervical arterial dissection
ICH	intracerebral hemorrhage
NIHSS	National Institutes of Health stroke Scale
mRS	modified Rankin Scale
tCeAD	traumatic CeAD
sCeAD	spontaneous CeAD
sICH	symptomatic ICH
tPA	tissue Plasminogen Activator
