# Supplementary material for: Biological Sex and Outcomes in Patients with Extracranial Cervical Arterial Dissections
Source: J Clin Med. 2025 May 29;14(11):3816. doi: 10.3390/jcm14113816 (PMC12156125; doi:10.3390/jcm14113816)
Supplement: Supplementary file 1 [file jcm-14-03816-s001.zip › jcm-3621669-supplementary.pdf]

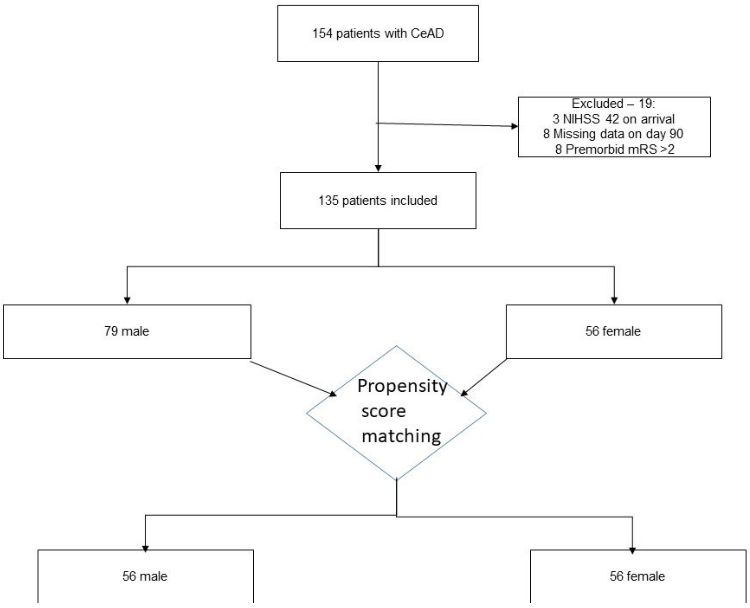

Supplementary Figure S1

Supplementary Table S1: Baseline characteristics in patients with CeAD and stroke based on Gender

	males (n=50)	Females (n=32)	p
Age, (median, IQR)	47.5 (40, 55)	43.5 (37, 54)	0.144
Hypertension (%)	15 (30)	6 (19)	0.255
Diabetes (%)	7 (14)	2 (6)	0.273
Atrial fibrillation (%)	3 (6)	1 (3)	0.555
Hyperlipidemia (%)	13 (26)	1 (3)	0.007
Ischemic heart disease (%)	2 (4)	0 (0)	0.252
Current Smoker (%)	17 (34)	5 (16)	0.067
Prior stroke (%)	2 (4)	1 (3)	0.837
Migraine (%)	0 (0)	0 (0)	1.00
Spontaneous dissection	40 (80)	28 (87.5)	0.379

Isolated neck injury (%)	6 (12)	1 (3)	0.161
Poly-trauma (%)	4 (8)	2 (6)	0.767
Trauma severity			0.576
Non trauma (%)	40 (80)	29 (91)	
Mild (%)	4 (8)	1 (3)	
Moderate (%)	1 (2)	0 (0)	
Severe (%)	5 (10)	2 (6)	
Pre-stroke mRS≤ 2 (%)	49 (98)	32 (100)	0.421
Admission NIHSS (Median, IQR)	4 (2, 15)	2 (0, 4)	0.010
Early infarct on imaging (%)	32 (64)	22 (69)	0.658
Involved vessels:			
Carotid only (%)	30 (60)	16 (50)	0.373
Vertebral only (%)	13 (26)	12 (37.5)	0.270
More than one vessel (%)	9 (18)	5 (16)	0.780
Radiological findings:			
Intimal flap	8 (16)	6 (19)	0.747
Pseudo-aneurism (%)	14 (28)	11 (34)	0.541
Mural hematoma (%)	14 (28)	8 (25)	0.765
Elongated stenosis (%)	19 (38)	18 (56)	0.105
Double lumen (%)	2 (4)	2 (6)	0.645
Occlusion above bifurcation (%)	21 (42)	10 (31)	0.327
Long tapering (%)	1 (2)	0 (0)	0.421
Flame shaped occlusion (%)	25 (50)	6 (19)	0.004
% Vessel stenosis			0.095
No stenosis	7 (14)	5 (16)	

<50%<br>50%-69%<br>70%-99%<br>100%	1 (2)<br>1 (2)<br>15 (30)<br>26 (52)	1 (3)<br>6 (19)<br>9 (28)<br>11 (34)	
tPA (%)	7 (14)	2 (6)	0.273
Stent placement (%)	28 (56)	13 (41)	0.174

IQR – interquartile range, mRS – modified Rankin Scale, NIHSS- National Institutes of Health Stroke Scale, tPA – tissue plasminogen activator.

Supplementary Table S2: Treatments and Outcomes in patients with CeAD and stroke based on Gender

	Males n=41	Females n=30	p-value
Any ICH (%)	4 (10)	1 (3)	0.388
sICH	2 (5)	1 (3)	1.000
Recurrent stroke	5 (12)	4 (13)	1.000
Recurrent dissection	2 (5)	0 (0)	0.506
Favorable Recanalization	21 (62)	11 (46)	0.288
mRS day 90 ≤2*	36 (88)	28 (93)	0.453

ICH – intracerebral hemorrhage, LMWH – low molecular weight heparin, NIHSS- National Institutes of Health Stroke Scale, mRS – modified Rankin Scale, sICH – symptomatic intracerebral hemorrhage.

Supplementary Table S3. Factors associated with favorable (mRS≤2) outcome at 90 days post stroke on logistic regression.

Variable/Group	OR	95% CI	p
Age (per year)	1.08	1.02-1.15	**0.008**
Biological sex (male)	0.53	0.09-3.20	0.490
Multiple arteries	0.05	0.01-0.41	**0.005**
Vessel occlusion	0.13	0.02-0.90	**0.039**
Admission NIHSS	0.87	0.77-0.96	**0.009**
sICH	1.55	0.08-31.34	0.780

NIHSS – National Institutes of Health Stroke Scale, sICH – symptomatic intracranial hemorrhage.
